# A new governance space for health

**DOI:** 10.3402/gha.v7.23507

**Published:** 2014-04-13

**Authors:** Ilona Kickbusch, Martina Marianna Cassar Szabo

**Affiliations:** 1 Global Health Programme, Graduate Institute of International and Development Studies, Geneva, Switzerland; 2 Mailman School of Public Health, Columbia University, New York, NY, USA

**Keywords:** global health, governance, diplomacy, global public goods for health

## Abstract

Global health refers to ‘those health issues which transcend national boundaries and governments and call for actions on the global forces and global flows that determine the health of people’. (Kickbusch 2006) Governance in this trans-national and cross-cutting arena can be analyzed along three political spaces: global health governance, global governance for health, and governance for global health. It is argued that the management of the interface between these three political spaces of governance in the global public health domain is becoming increasingly important in order to move the global health agenda forward. **Global health governance** refers mainly to those institutions and processes of governance which are related to an explicit health mandate, such as the World Health Organization; **global governance for health** refers mainly to those institutions and processes of global governance which have a direct and indirect health impact, such as the United Nations, World Trade Organization or the Human Rights Council; **governance for global health** refers to the institutions and mechanisms established at the national and regional level to contribute to global health governance and/or to governance for global health – such as national global health strategies or regional strategies for global health. It can also refer to club strategies, such as agreements by a group of countries such as the BRICS. In all three political spaces, the involvement of a multitude of state and non-state actors has become the norm – that is why issues of legitimacy, accountability and transparency have moved to the fore. The transnational nature of global health will require the engagement of all actors to produce global public goods for health (GPGH) and to ensure a rules-based and reliably financed global public health domain.

This article aims to set out the transnational and cross-cutting nature of governance in the global public health domain along three political spaces: global health governance, global governance for health, and governance for global health. The reform and strengthening of governance institutions in all three political spaces as well as their interface is critical to keep global health firmly on the political agenda, to strengthen action on the determinants of health, and to ensure that governance is accountable and transparent to those who have a stake in its viability and legitimacy. When addressing issues of governance, we consider it more precise not just to speak about ‘global health’, but to conceive of the arena of the multitude of public and private actors and competing interests as a ‘global public health domain’ (1), within which many hubs for networking and negotiation have emerged (1).

Governance in the global public health domain must grapple with six key challenges that present overlapping opportunities for policy innovation and institutional development: How can competing interests and fragmentation be overcome? How can a greater commitment by countries for providing global public goods for health (GPGH) be ensured? What role should corporations and their foundations play in global health, and how can the private sector become more accountable? How can reliable funding be ensured for global health initiatives and organizations? How can political support be gained for addressing the political, social, and commercial determinants of global health? How can the voice of civil society be ensured in global health governance? The transnational nature of global health will require a focus on providing GPGH and engaging these different actors to support a rules-based and well financed global public health domain.

## Defining global health and global governance

The understanding of global health governance is contingent on the definition of global health, several of which have been proposed. Battams and Matlin discuss the implications of several different attempts to define the scope and purpose of global health, highlighting the range of goals and institutions involved in the global health arena (2). Debate tends to focus on understanding the relationship between public health and global health, and on the inclusion of value-based concepts, such as health equity, in the definition of global health activities. However, all definitions in some way acknowledge the rise of global interdependence that has come to characterize global health. Considering the field of global health as it relates to global governance, Kickbusch (3) defines ‘global health’ to refer to ‘those health issues which transcend national boundaries and governments and call for actions on the global forces and global flows that determine the health of people. It requires new forms of governance at national and international levels which seek to include a wide range of actors’ (3). This definition aims to encompass three key characteristics: the global nature of the issue, the importance of transborder determinants of global health, and the global governance actions that are required. Global health is essentially characterized by new multi actor approaches that aim to deal with global interdependence as well as new power relationships; for example, the global forces and global flows that determine health can no longer be resolved by one nation or sector but can be significantly shaped by one industry – as is the case with tobacco.

**Fig. 1:**
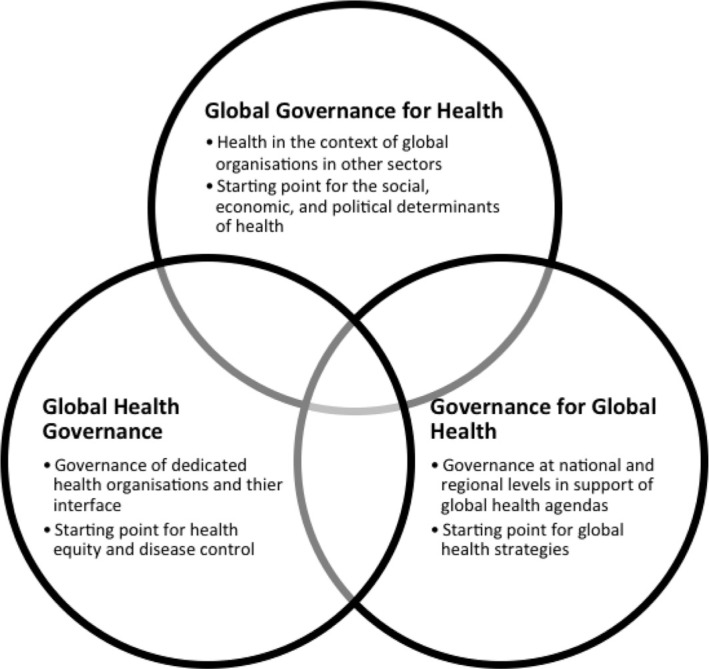
Global health governance along three political spaces.

Many definitions of **global governance** also put interdependence in the center. One of the most classic is Rosenau who defines it as a ‘purposive order for the management of interdependence in the absence of a global state’ (4). Global governance with a purpose implies a system of rules, processes and institutions which functions and operates at the global level and provides the frame within which actors interact and take decisions on priorities and direction. Fidler closely follows this when he defines **global health governance** as referring ‘to the use of formal and informal institutions, rules, and processes by states, intergovernmental organizations, and non-state actors to deal with challenges to health that require cross-border collective action to address effectively’ (5). However, given the interplay between actors and interests at the domestic and international levels, it can prove helpful to analyze global health governance along three political spaces in order to fully appreciate the links and the interface between the different institutions and processes involved in global health. (See Fig. 1) **Global health governance** refers mainly to those institutions and processes of governance with an explicit health mandate, such as the World Health Organization (WHO); **global governance for health** refers mainly to those institutions and processes of global governance which do not necessarily have explicit health mandates, but have a direct and indirect health impact, such as the United Nations, the World Trade Organization or the Human Rights Council; **Governance for global health** refers to the institutions and mechanisms established at the national and regional level to contribute to global health governance and/or to governance for global health – such as national global health strategies or regional strategies for global health.

Just as public health challenges can no longer be effectively addressed only within the health sector and at the national level, the WHO can no longer be the sole manager of intergovernmental challenges relating to the governance of global health. Morrison (6) termed the last two decades as the ‘golden era of global health’, as a result of the explosion of actors in the global health arena, along with dramatic increases in funding for global health initiatives on a range of issues (6). However, as the ‘golden era’ has evolved, so too have the challenges facing global health: no longer is the global health arena primarily focused on technical, medical, and professional problems and solutions; it has gained in political and commercial relevance and is therefore much more subject to political and commercial interests. And – as WHO Director General Dr. Chan has expressed – ‘market power readily translates into political power. Few governments prioritize health over big business’ (7). The challenges facing the ever-expanding global public health domain are therefore less of a technical nature – in many areas we already have the knowledge and the technologies – but require political will and the willingness of states and other actors to prioritize health. That is why there must be more concern with the political and commercial determinants of health.

The ‘golden era of global health’ led to an explosion of players in the global health arena and, in particular, resource strong non-state actors have grown significantly in influence. They can set large parts of the global health agenda independently, create or close organizations, and exert political pressure on donor and recipient countries often without accountability, transparency, or coherence with other institutions. At the end of the 66th World Health Assembly (WHA66) in May 2013, WHO Director General Dr. Chan stated that the pressure from trans-national private companies on Member States was never stronger, particularly in relation to the non-communicable disease (NCD) agenda, comparing it to earlier times on matters such as essential medicines. She further emphasized this in her speech to the Global Health promotion Conference in Helsinki: ‘Efforts to prevent NCDs go against the business interests of powerful economic operators. In my view, this is one of the biggest challenges facing health promotion (…) Public health must also contend with Big Food, Big Soda, and Big Alcohol. All of these industries fear regulation, and protect themselves by using the same tactics’ (7).

**Fig. 2:**
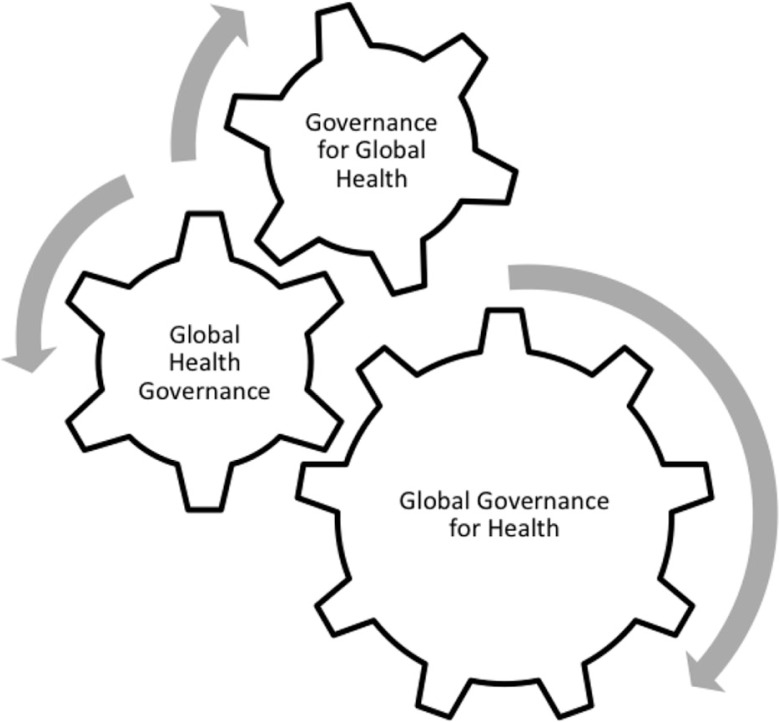
Linking actors within and between dimensions.

As the global health industry grows to represent over 1/8 of global economic flows, it is essential that global health governance institutions firmly establish processes to link actors within and between sectors and define firewalls and conflict of interest strategies. (See Fig. 2) The collective problem solving required in the global public health domain requires these controversial actors to be involved but without a commonly agreed rule based system for including non-state actors in global governance institutions, it is difficult to subject these powerful organizations – large corporations, foundations and NGOs – to critical analysis. This is the challenge that now faces the WHO as it sets up the United Nations Interagency Task Force on the Prevention and Control of NCDs.

## Global health governance

*Global health governance* refers mainly to those institutions and processes of governance that have an explicit health mandate, such as the WHO, hybrid organizations such as the GAVI Alliance (GAVI) and the Global Fund to Fight AIDS, Tuberculosis, and Malaria (GFATM), as well as health focused networks and initiatives and non-governmental organizations. Many of these organizations are located in Geneva – which is sometimes referred to as the ‘capital of global health’. The architecture of this dimension of the global public health domain has been much analyzed, with some viewing WHO in the center of the arena (based on its constitutional mandate) and others seeing a more polycentric structure emerging in which the WHO is just one of many players in global health governance. The expansion of the actors and institutions with influence in the global public health domain has clearly led to new power relationships. The hybrid organizations have introduced constituency based models of governance, whereas state based international organizations such as the WHO are becoming subject to increased scrutiny, especially regarding conflicts of interest. Consequently – as a part of the WHO reform – the WHO will propose a new framework for working with non-state actors, both from the NGO and civil society arena and from the private business sector.

Global health has been an arena of governance innovations in legislation, organizational structure, financing, and cooperation, and governance institutions such as GFATM and GAVI are emblematic of a new ‘constituency model’ of global health governance. The constituency model of governance coordinates representatives from government, private foundations, civil society, private sector, multi and bilateral organizations, and affected persons with the aim of developing harmonized structures to work for health. So-called ‘public-private partnerships’ with a health focus serve to define global health issues and provide a forum for actors from all three governance political spaces to network and negotiate global health priorities. Given that GFATM has approved roughly USD 30 billion of funding for different health programmes, innovative organizations in this dimension of governance can marshal significant resources and involve a vast range of participants, sectors, and collaboration strategies, with the aim of setting health priorities and moving health issues into other sectors.

It is often disregarded that organizations explicitly concerned with global health governance usually have several mandates. They engage in setting rules, norms and standards, they act as network hubs for public, private and civil society actors engaged around specific issues, they promote and frame health agendas and provide for health at the country level through global health initiatives and funding mechanisms. However, there is an increasing convergence that has not yet been thoroughly analyzed: these organizations all bring health onto the agendas of other institutions engaged in global governance, particularly global summits like the Rio +20, the UN General Assembly, the G8, or the G20. Global health governance requires the constant ‘vertical’ exchange between engaged actors from the national, regional and global levels, and ‘horizontal’ exchange between institutions and organizations with very different goals and stakeholders – indeed an extraordinary challenge for network governance.

Global health governance is still defined by the fact that it is one of the few global issue areas with its own dedicated international agency with treaty-making power. The WHO continues to serve as the main global health governance venue for legitimate decision-making processes, and it also serves an essential and unique coordinating role. With the adoption of the Framework Convention on Tobacco Control in 2003, the revision of the International Health Regulations in 2005, and the adoption of other frameworks and codes on global health issues such as virus sharing and mobility of health workers, WHO has regained relevance. Indeed, there are regular calls for increased legislative activity: most recently a Global Framework Convention on Research and Development was proposed. Other legislative suggestions include treaties and codes in relation to alcohol, marketing to children, falsified medicines, and anti microbial resistance. There are also suggestions for an all-encompassing Framework Convention on Global Health.

Providing an interface for what Wiseman has termed ‘polylateral diplomacy’ within the formal WHA processes is one of the governance challenges faced by WHO. Suggestions, such as the proposal for a ‘Committee C’ to address policy coherence and accountability, have been put forward for mechanisms to support normative and strategic coordination among different actors in global health. As the global public health domain has expanded, not only has the formal WHA agenda become overloaded, member states and other actors now increasingly use the WHA as a forum to discuss issues that are not on the agenda. At the WHA66, dozens of such events were organized throughout Geneva by member states as well as other health organizations and the private sector, often with overflowing attendance, reflecting the priorities and positioning of member states and attendant NGOs, private-sector actors, and other attendees. They included issues such as antimicrobial resistance (AMR), women's and children's health, response to H7N9, universal health coverage, global health diplomacy, eHealth and health internet domain names, and neglected tropical diseases. These meetings do not only reflect the need to discuss issues in a format that is different from the WHA process, where formal statements by delegates restrict what can be said, but they also provide ministers of health a platform on which they can position themselves more proactively and politically than in the formal WHA sessions.

These side events reflect the convening power of WHO as a network and negotiation hub that can support and strengthen the other two political spaces of global governance. By providing a site for national delegations to raise challenges of domestic concern, WHO serves as an arena to define and prioritize global health issues. Once these issues have been brought to the forefront of the agendas of organizations within the realm of global health governance, they can be incorporated into the institutions and processes of global governance for health, such as the UN, WTO, and World Bank. A recent example of such a multi-facetted process involves the shaping of the NCD agenda. This began at least a decade ago with health experts drawing attention to NCD issues and was followed by resolutions and action plans at the WHO; the NCD agenda then moved to the United Nations and was finally debated there in 2011 by heads of governments; after being recognized at UNGASS as a global priority, it was referred back to the health ministers at WHO to set priorities and indicators; finally – after reaching agreement at the WHA 2013 – NCDs were debated at ECOSOC 2013. Here, a resolution was adopted to establish a WHO-led Interagency Task Force on the Prevention and Control of Non-Communicable Diseases. During this process, a multitude of actors were engaged to drive the agenda forward at different negotiation hubs. This is an interesting contrast to the 1990s, when countries did not think WHO capable of handling the interagency dimensions of the HIV/AIDS pandemic and created UNAIDS to address the issue.

Other agendas are in the wings to be taken forward in a similar manner: Efforts to address the growing issue of AMR began roughly 15 years ago, when WHO convened a series of expert meetings, culminating with a series of recommendations and strategies published in 2001; a handful of countries such as Denmark took up the issue as a matter of national priority, developing governance strategies such as the Danish Integrated AMR Monitoring and Research Programme (8) and establishing national guidelines for use; the AMR issue was again highlighted by WHO in 2011, designated as the focus of that year's World Health Day and named as an organization-wide priority; at the WHA66, a side event was organized with overflowing attendance, leading to member states requesting the AMR issue be a priority in 2014 discussions. It is hoped that the AMR issue will now move onto the agenda of the United Nations and other global governance institutions.

## Global governance for health

*Global governance for health* refers mainly to those institutions and processes of global governance that do not necessarily have explicit health mandates, but that have a direct and indirect health impact, such as the World Trade Organization and the post-2015 MDG/Sustainable Development Goal (SDG) process. Many of these institutions and processes are related to the social determinants of health, and to the global flows of goods, services, and ideas related to health. Increasingly – like the United Nations General Assembly – they set health agendas. As global health issues and actions become more crosscutting between sectors, actors, institutions, and processes as a result of globalization, the majority of progress in global health is likely going to come from institutions and processes relating to global governance for health. Several authors, such as Frenk and Moon, have discussed the range of policymaking arenas that influence the global health system, including international trade, security, migration, and the environment (9). Global health is also enjoying an increased role in global organizations traditionally focused in other sectors, such as the ILO, FAO, and WIPO. It is in this dimension that global health governance and governance for global health can be supported and propelled forward, if health issues are well defined (by dedicated health organizations) and advocated (by national, regional and global actors).

Rio+20, or the United Nations Conference on Sustainable Development, is emblematic of how the global governance for health dimension can serve as the basis for advancing global health: this dimension of global governance is concerned with the starting points of social, economic, and political determinants of health. The Conference convened world leaders and government officials, private sector representatives, heads of global organizations, and representatives of civil society to discuss sustainable development in seven areas: jobs, energy, cities, food, water, oceans, and disasters. Over USD 513 billion was pledged towards working for the ‘Future We Want’, by building green economies, eradicating poverty, and improving international coordination on development issues. Global health plays a clear role in the achievement of these aims; the Rio+20 report emphasized that, ‘health is a precondition for and an outcome and indicator of all three [economic, social, and environmental] political spaces of sustainable development’ (10).

In the post-2015 MDG/SDG development process, the United Nations has once again been the institution to take the lead in responding to a major question of global governance. The UN Secretary-General launched a High-Level Panel of Eminent Persons (HLP) in July 2012, which produced a report 10 months later (11). This report contains a proposed, ‘Goal 4: Ensure Healthy Lives’, which continues the original MDG priorities and expands them to include sexual and reproductive health and rights, but does not reference some of the most pressing global health challenges with GPGH opportunities, such as AMR and outbreak crises. Other work streams are contributing to the definition and forging of the Post-2015 Goals negotiations, including a 30-member Open Working Group of the General Assembly, and the UN System Task Team, the latter of which produced a report entitled ‘The Future We Want’, in which health is not one of six major goals, but plays a clearly cross-cutting role as a subcategory of several domains such as human rights, peace and security, and social development. Despite the relatively low-profile given to explicit health goals in the HLP report, it is through proposals such as this that advocates at the national level can propel the global health agenda into the priorities of other global organizations and governance projects.

Similar to the post-2015 process, discourse surrounding the ideal structure and financing of development aid is evolving within the context of this dimension of global governance. As global organizations take up questions of food security, equity, poverty reduction, human rights, financial stability, and environmental stewardship, health goals have clearly crosscutting relevance. As emphasized in the Rio+20 report, health is not only affected by cross-cutting global governance arenas such as food and water security and institutional development, but it may support or undermine these other governance challenges if not effectively addressed. These challenges increasingly relate to patterns of production and consumption.

This dimension of global governance for health debate is linked to the challenge of the NCDs and the increasing influence of transnational companies and their products on population health. This refers back to the statements by the Director General of the WHO at the Global Conference on Health Promotion in Finland quoted above that, ‘efforts to prevent NCDs go against the business interests of powerful economic operators’ (7). Countries will need to explore collective action through global mechanisms and instruments – such as the Framework Convention in Tobacco Control – to address these drivers of NCDs at both the national and the international levels. These drivers have been termed the commercial determinants of health, and they have been defined as ‘the factors that influence health which stem from the profit motive’ (12). The role of civil society will be critical to address the many sectors of policy that need to act in response to these challenges by addressing critical health issues and counteracting the impact of lobbying. A recent report by The Credit Suisse Research Institute highlights the example of the negative impact of sugar consumption on health, and indicates directions for future policy response (13) most of which lie outside of the action realm of the health sector and transcend the national level.

## Governance for global health

*Governance for global health* refers to the institutions and mechanisms established at national and regional levels that contribute to global health governance and/or to governance for global health. The changing nature of global health makes this form of governance ever more crucial, as national and regional mechanisms must both support and respond to global governance institutions and processes. As a growing industry, the health sector represents over USD 6.5 trillion of global flows, and this figure is projected to surpass 10 trillion by 2020. These interests, public and private, exert enormous pressures on governance mechanisms at the domestic level. The health sector and the domestic institutions and process that contribute to global health – ranging from outbreak surveillance mechanisms to insurance systems, and more broadly to financial stability and food security – is a critical issue in domestic politics around the world. Given the crosscutting nature of global health, national and regional governance for global health must have strategies firmly in place for navigating the intersection between domestic and global interests and politics.

Slaughter notes the importance of understanding domestic issues in their contexts of regional and global policy networks, if the intersection of national and global health policy is to be effectively addressed (14). National solutions to domestic issues depend increasingly on regional and global contexts and multi-sectoral resources. Simultaneously, when national delegations – be they ministers of health, of foreign affairs, or other diplomats– approach the spheres of global health governance and global governance for health, they are bound by domestic political interests and priorities. Optimal governance solutions will depend on aligning national priorities and global responsibilities, such that domestic institutions can support governance for global health with strategies for policy coherence and inter-sectoral cooperation. This relates to concepts such as ‘Healthy Public Policy’, which emphasizes the need to consider health as a shared value across all sectors. For example, domestic governance questions, such as the aging of societies, social inequities, and financial stability, are all determinants of and influenced by health. Given that health has important effects on goals of other sectors, ‘Healthy Public Policy’ aims to harmonize policies across sectors, to complement public health initiatives and ensure coordination between ministries and diplomats. Increasingly, this also includes foreign policy.

Several countries have taken the lead on developing national strategies for governance for global health. In 2007, Switzerland established the Swiss Health Foreign Policy, developed by the Departments of the Interior and the Department of Foreign Affairs to integrate national and global health policies. The UK's ‘Health is Global’ strategy, launched first in 2008, aims to manage the relations between different government departments, supporting cooperation and policy coherence domestically, regionally, and globally. In the United States, the Council on Foreign Relations, the Departments of Defense and of State, and the Center for Strategic International Studies (CSIS) have all taken up the question of global health policy, analyzing domestic opportunities to strengthen global health diplomacy and support polylateral coordination of health programs. National institutions such as these are critical to understanding the crosscutting policy spaces relating to global health: the intersection between global and domestic sectors and institutions, between human rights and global public goods, and between public and private interests, to name but a few challenging arenas that governance for global health must be prepared to navigate.

Kickbusch has emphasized that, ‘global health begins at home’, with the attitude that health goals should be shared across domestic sectors and government agencies, and that all diplomats should work to achieve health goals, irrespective of their governance context (15). Health Ministers must now be concerned with the priorities and activities of the security, trade, finance, agriculture, development, and employment industries if they are to effectively address health issues domestically and in global negotiations. As the role of Ministries of Health changes in the sphere of global health governance, so too does the role of foreign ministries operating in the dimension of global governance for health. Domestic policies taking a ‘Healthy Public Policy’ approach seek to integrate health goals across all levels of governance, convincing negotiators outside the health sector to speak on health in other arenas. This approach also necessitates taking the goals developed at the level of global governance for health, such as the MDGs or the post-2015 SDG process, and implementing them on a national level across policy areas.

## Conclusions

We need to gain a better understanding of the interface between the three dimensions of governance in the global public health domain. All three are highly relevant and managing them well can support the progress of public health in a global environment. All three are driven by contestations of power, interests, and values, which in turn translate into political and commercial determinants of health. Health is back on the political agenda and the health debate at this point in time is fundamentally about the definition of the common good and the role of the state, the market, and the community in a period of globalization, commercialization and individualization. GPGH can only be produced if all three political spaces for governance combine to serve this purpose.
